# Supplementary material for: Survey on Limnic Gastropods: Relationships between Human Health and Conservation
Source: Pathogens. 2022 Dec 13;11(12):1533. doi: 10.3390/pathogens11121533 (PMC9786810; doi:10.3390/pathogens11121533)
Supplement: Supplementary file 1 [file pathogens-11-01533-s001.zip › pathogens-1982681-supplementary.pdf]

Supplementary Materials:

Table S1: Distribution of limnic gastropods and associated trematodes and their coordinates

Collection point data			Limnic gastropods											Larval trematodes		
			Ampullariidae	Planorbidae							Succineidae	Lymnaeidae	Physidae			
Points	Geographic coordinates UTM	Hydrographic units	*Pomacea* sp.	*Biomphalaria* sp.	*B. glabrata*	*B. straminea*	*B. tenagophila*	*B. cousini*	*B. kuhniana*	*Drepanotrema cimex*	*Omalonyx* sp.	*Pseudosuccinea columella*	*Stenophysa marmorata*	Echinostome	Xiphidiocercaria	Strigeid ocercaria
1	-18,775347<br>-43,255115	HU1		X	X				X				X			
2	-18,773126<br>-43,269246	HU1		X	X				X							
5	-18,77955<br>-43,285942	HU1	X	X	X	X			X	X			X			
6	-18,792883<br>-43,289729	HU1		X	X								X			
9	-18,760984<br>-43,342033	HU3		X	X								X			
10	-18,759874<br>-43,347945	HU4		X	X							X				
11	-18,734675<br>-43,361932	HU5		X					X	X			X			X
12	-18,737719<br>-43,363778	HU5		X									X			
15	-18,730363<br>-43,365636	HU4		X	X					X			X			
18	-18,763129<br>-43,413238	HU7									X	X	X			
25	-18,78412<br>-43,377884	HU7											X			
32	-18,858279<br>-43,338465	HU9		X						X			X		X	X
33	-18,856991<br>-43,339102	HU9		X			X						X	X		
37	-18,697598<br>-43,332415	HU12		X	X											
39	-18,707207<br>-43,336425	HU12											X			
40	-18,721655<br>-43,33313	HU11		X				X								
41	-18,72545<br>-43,32192	HU1	X	X	X				X				X			

Table S2: Jaccard's analysis for hydrographic units

	HU1	HU3	HU4	HU5	HU7	HU9	HU12	HU11
HU1	1							
HU3	0.42	1						
HU4	0.5	0.6	1					
HU5	0.57	0.4	0.5	1				
HU7	0.1	0.2	0.33	0.16	1			
HU9	0.37	0.4	0.5	0.6	0.16	1		
HU12	0.42	1	0.6	0.4	0.2	0.4	1	
HU11	0.12	0.25	0.16	0.2	0	0.2	0.25	1

Table S3: Jaccard's analysis for the limnic gastropods

	Biomphalaria sp.	*B. glabrata*	*B. straminea*	*B. tenagophila*	*B. cousini*	*B. kuhniana*	*S. marmorata*	*D. cimex*	*Omalonyx* sp.	*P. columella*	*Pomacea* sp.
Biomphalaria sp.	1										
B. glabrata	0.57	1									
B. straminea	0.14	0.25	1								
B. tenagophila	0.14	0	0	1							
B. cousini	0	0	0	0	1						
B. kuhniana	0.28	0.2	0.5	0	0	1					
S. marmorata	0.75	0.57	0.14	0.14	0	0.28	1				
D. cimex	0.57	0.3	0.25	0.25	0	0.5	0.57	1			
Omalonyx sp.	0	0	0	0	0	0	0.14	0	1		
P. columella	0.12	0.2	0	0	0	0	0.28	0.2	0.5	1	
Pomacea sp.	0.14	0.25	1	0	0	0.5	0.14	0.25	0	0	1

Table S4: Logistic regression for *Biomphalaria glabrata*

Variable Independent	P	OR
B. tenagophila	Collinearity	
B. straminea	Collinearity	
Biomphalaria	Collinearity	
B. cousini	Collinearity	
B. kuhniana	Prob > chi2 = 0.0015	28.8
Drepanotrema	Prob > chi2 = 0.1488	
Omalonyx	Collinearity	
Pseudosuccinea	Prob > chi2 = 0.3222	
Stenophysa	Prob > chi2 = 0.0064	8.5
Pomacea	Collinearity	
